# Prospects of sustainable polymers

**DOI:** 10.1038/s41598-024-59439-z

**Published:** 2024-04-24

**Authors:** Anuj Kumar, Vijay Kumar Thakur, Hamed Yazdani Nezhad, Kwan-Soo Lee

**Affiliations:** 1 School of Materials Science and Technology, Indian Institute of Technology (BHU), Varanasi, 221005 India; 2 Biorefining and Advanced Materials Research Centre, Scotland’s Rural College, Edinburgh, EH9 3JG UK; 3 School of Mechanical Engineering, University of Leeds, Leeds, LS2 9JT UK; 4 MPA-11: Materials Synthesis & Integrated Devices, Los Alamos National Laboratory, Los Alamos, NM 87545 USA

**Keywords:** Sustainable polymers, Bio-based polymers, Fossil-based materials, Circular economy, Materials science, Environmental social sciences

## Abstract

Synthetic polymers have shown a great impact on every aspect of our life and attained an exponential rise in their production and utilization in the past decades due to their durability, flexibility, moldability, and inexpensive nature. However, the use of natural polymers or development of safe and environment-friendly synthetic bio-based polymers is continuously undergoing for a sustainable future owing to the exhaustion of petroleum-based resources or fossil-based materials, disposal and economical concerns, including government guidelines. In this regard, the development of new sustainable polymers or materials will step up and build a genuinely circular economy by decreasing manufacture or utilization of fossil-based materials as limited reserves.

Polymers are essential in various aspects of our daily life due to their low-cost, durability, flexibility, and moldability, but their huge and unbalanced manufacturing processes and non-degradability behavior have put a burden by polluting the environment due to their single-use and throw them anywhere. Currently, it is impossible to remove the use of synthetic polymers completely, but they can be avoided in some ways or seeking out alternatives to overcome this plastic polymers ecosystem for better and sustainable future prospects. Therefore, the researchers are focusing to find ways to develop more sustainable alternatives via different modified processes of feedstock acquisition, material design and fabrication, use and recycling of the used plastics to monomers. The research activities for these sustainable polymers might start from renewable feedstock, using green methods and renewable energy, measurement of environmental and economic impacts^1,2^. In this way, renewable resources (e.g., agricultural and food wastes, woody materials, recycled products) are extensively used up for synthesizing or developing new polymers, monomers or forms for variety of application in industry and biomedical areas^3–5^. Sustainable polymers, derived from renewable and waste materials and their mixtures, can be biodegraded, composted or recycled. They present lessened environmental effects during their life phases. Nowadays, these sustainable polymers are getting favorable attention and potential utilization in diverse applications in industrial and biomedical areas^2,6^.

## Collection overview

Sustainable polymers have already found an increasing interests and utilization in diverse applications worldwide. The direct focus of finding sustainable alternatives is to uphold the benefits of plastic materials while decreasing their undesirable effects. However, the complete replacement of these synthetic plastic materials by developing new sustainable polymers is of utmost priority of the researchers for a sustainable future^1,7^. In this special issue of *Sustainable Polymers*, prominent researchers present advances in some of these central areas described above and share their cutting-edge research activities. Abdel‑Baky et al*.* designed and developed a new sustainable and multifunctional chitosan-quinoline (CHQ) Schiff base derivative containing effective antibacterial, antioxidant, and antidiabetic accomplishments to speed up wound healing process specifically in diabetic patients. In this study, molecular docking simulation was employed to show the clarification of binding modes of this derivative for inhibiting α-amylase and α-glucosidase and using as potential diabetes mellitus drug for regulating the blood glucose level, while DFT computational analysis was utilized to elucidate the charge density distributions theoretically correlated to its biological activity^8^. In another study, Gierszewska et al*.* developed adenine-modified edible chitosan (CH) films involving the mixture of choline chloride and citric acid as plasticizer. In this study, an impact of adenine (A, vitamin B4) content on the functional properties of the developed films was assessed. These films were water-insoluble and showed enhanced antioxidative (55.8–66.1% of H_2_O_2_ scavenging action) and non-mutagenic (lack of growth of Salmonella typhimurium) properties, lessened oxygen transmission, good antimicrobial and mechanical characteristics^9^. This study demonstrated the efficacy of the developed materials as edible coating for food packaging applications.

The extensive use of synthetic polymers, like polyethylene terephthalate (PET), and their non-degradable behaviors showed the increasing problem of plastic wastes, leaking into the landfills, environment, and oceans as fragments of micro- and nano-plastics. Therefore, Rashwan et al. demonstrated the utilization of recycled PET (rPET) as sustainable value-added material feedstock for material-extrusion additive manufacturing technology. For this, rPET was compounded with low-cost additives such as pyromellitic dianhydride (PMDA, a chain extender), maleic anhydride-functionalized styrene-ethylene-butylene-styrene terpolymer (MA-g-SEBS, a thermal modifier/toughening agent), ethylene-ethyl acrylate-glycidyl methacrylate terpolymer (E-EA-GMA, a reactive elastomeric impact modifier), and ethylene-ethyl-acrylate (EEA, non-reactive elastomeric impact modifier), and extruded into filaments using twin-screw extruder for potential application in prototyping, tooling and testing parts, or end-use internal parts of small machines and cars^10^. In another approach, Verma et al*.* developed a polyurethane (PU)-based coating material by incorporating multifunctional SiO_2_@ZnO core–shell nanospheres (4 wt%) for achieving durability and sustainability in marine transport. This polymeric coating showed a transition from hydrophilic to hydrophobic state (⁓125.2° ± 2°) and demonstrated remarkable surface abrasion resistance, enhanced antibacterial (⁓100%), antifungal (⁓95%), and antialgae (⁓90%) properties compared to only PU for using it ideally to protect steel surfaces against biofouling^11^. In an approach, Trakunjae et al*.* established the statistical optimization of the higher production of poly(3-hydroxybutyrate-co-3-hydroxyhexanoate) [P(3HB-co-3HHx)] bacterial copolymers (a next-generation bioplastic) by *Cupriavidus necator* PHB-4/pBBR CnPro-*pha*C_Rp_ using response surface methodology (RSM) and evaluated for its characteristics. The characteristics of this produced polymer were similar to marketable P(3HB-co-3HHx) and exhibit its suitability for a broad range of applications^12^.

Conjugated polymers are considered as favorable tools to distinguish different types of semiconducting single-walled carbon nanotubes, but the synthesis of these polymers is a challenging task due to inadequate control over molecular weights and unpredictive/unrepeatable batches hamper scaling-up and potential applications. Additionally, commercial catalysts (homogenous) frequently need conditions and nearly impossible to recycle. To overcome these problems, Wasiak et al*.* developed a heterogeneous nanocatalyst comprising of magnetic nickel nanowires (NiNWs) enriched with greatly active palladium nanoparticles (PdNPs) as a welding tool for synthesizing polyfluorene derivatives by Suzuki polycondensation of fluorine-based monomer under microwave radiation that helped to decrease the reaction time from 3d to 1 h. The catalytically synthesized polymer derivatives were utilized to extract specific s-SWCNTs to validate their usefulness^13^.

In many industrial areas, fibre-reinforced polymers (FRP) are widely utilized and helped in reducing CO_2_ emissions owing to their remarkable characteristics in lightweight design. However, some used materials (e.g., petroleum-based curing agents) in designing of FRP tend to show harmful effects to the environment and health. Therefore, it is necessary to develop a sustainable alternative. Therefore, Walter et al*.* developed fully bio-based curing agents (i.e., amino acids as biomolecules) as sustainable alternatives for epoxy resins over environmentally harmful petroleum-based curing agents (e.g., amine-based curing agents) and demonstrated the improved thermo-mechanical characteristics through thermokinetic methods, which remarkably lessen the negative aspects of epoxy composites^14^.

In conclusion, sustainable polymers offer a feasible resolution to address key issues caused by unbalanced exercises in synthetic polymers. Researchers are continuously working in developing new sustainable polymers and materials by considering their complexity in recycling and composting, on-demand fabrication scaling-up, reproducibility, and pricing for a circular economy strategy towards future sustainability^15,16^. However, there are still various challenges or limitations in terms of energy cost, selectivity in recycling or depolymerization, recyclability, functional stability. Therefore, extensive research activities are required to focus on these challenges by designing new monomers, polymer constructs, and more green chemistry or sustainable protocols as well as energy-efficient methods for their production and recycling of polymer wastes or composites.
